# The effect of host age and inoculation dose on infection dynamics of *Angiostrongylus vasorum* in red foxes (*Vulpes vulpes*)

**DOI:** 10.1186/s13071-016-1940-4

**Published:** 2017-01-03

**Authors:** Pia Webster, Jesper Monrad, Christian M. O. Kapel, Annemarie T. Kristensen, Asger L. Jensen, Stig M. Thamsborg

**Affiliations:** 1 Department of Veterinary Disease Biology, Faculty of Health and Medical Sciences, 100 Dyrlægevej, Frederiksberg C, DK-1870 Denmark; 2 Department of Plant and Environmental Sciences, Faculty of Science, Section for Organismal Biology, 40 Thorvaldsensvej, Frederiksberg C, DK-1871 Denmark; 3 Department of Veterinary Clinical and Animal Sciences, Faculty of Health and Medical Sciences, University of Copenhagen, 16 Dyrlægevej, Frederiksberg C, DK-1870 Denmark

**Keywords:** *Angiostrongylus vasorum*, Red fox, *Vulpes vulpes*, Host age, Infection dose, Worm counts

## Abstract

**Background:**

Infections and clinical cases of *Angiostrongylus vasorum* in dogs are found increasingly across Europe, thus rendering knowledge on its infection biology more important. We used red foxes as a carnivore model to examine the effect of host age and infection dose on the establishment of adult *A. vasorum* in single experimental infections.

**Methods:**

Fourteen juvenile and fourteen adult red foxes, free of metastrongyloid infections, were given a low (50) or high (200) dose of third-stage larvae (L3) of *A. vasorum*. Two groups of three foxes of each age group served as uninfected controls. Larval excretion by Baermann and blood parameters were followed for ten weeks. Worm counts were performed at necropsy by sequential perfusion, dissection and Baermann method.

**Results:**

The establishment rate (i.e. recovery in percentage of inoculation dose) of *A. vasorum* primary infections in red foxes was associated with host age and inoculation dose. In the low dose juveniles, 61% (range 52–72%) of the infection dose was recovered as worms in the pulmonary arteries and heart at necropsy while only 35% (21–50%) were recovered in the high dose. Corresponding establishment rates for adults were 39% (18–98%) and 8% (1–21%). In juveniles, a higher dose resulted in significantly higher adult worm counts, higher larval excretion and more pronounced pathophysiological changes, particularly in coagulation parameters. Earlier onset of patency was also found in the juveniles. In contrast, the larval excretion in high dose adults was very low and two infected animals never reached patency. However, a few adults showed only limited resistance as judged by excretion of larvae. The increase to very high larval excretion levels (> 4,000 larvae per g of faeces) after several months in a single animal, indicated that any potential acquired immunity does not affect worm fecundity.

**Conclusions:**

Resistance to a primary *A. vasorum* infection was generally higher in older animals, and this age resistance was reflected in lower worm counts and reduced excretion of larvae. The juvenile red foxes were fully susceptible, as reflected in high establishment rates. Although severe clinical disease was never observed in the foxes, *A. vasorum* infections in red foxes appear to be chronic and moreover, to resemble infections in dogs. The results underline the red fox as a suitable model as well as natural reservoir for the parasite.

## Background

The parasitic nematode *Angiostrongylus vasorum* is widely distributed in the temperate and subtropical zone where it infects domestic dogs and other canids, e.g. red foxes (*Vulpes vulpes*) which are considered to represent an important wildlife reservoir [1–4]. The adult nematodes are located in the pulmonary arteries and in the right ventricle of the heart where they produce eggs that are carried to the arterioles of the lung. After hatching, the first-stage larvae (L1) penetrate the alveoli, enter the bronchial system and are coughed up, swallowed by the host and finally excreted with the faeces. Gastropod intermediate hosts become infected by means of coprophagic behaviour, or penetration of the foot, and infective third-stage larvae (L3) develop in their tissue. The life-cycle is completed when a new canid host ingests infected snails or slugs [5, 6] or eventually paratenic hosts such as frogs or birds [7, 8]. Infected dogs may present with severe clinical signs caused by pneumonia and/or interference with blood coagulation, manifesting mainly as coughing, dyspnoea, fatigue, exercise intolerance, depression, bleeding disorders and subcutaneous swellings, but also with neurological signs, diarrhoea and vomiting, and possibly death [9–11]. An increased number of reports of *A. vasorum* infections in dogs and red foxes indicate that the geographical distribution of the parasite is expanding and that, due to the overall severity of the infection in dogs, *A. vasorum* is regarded as a significant and emerging veterinary problem in Europe [12].

Experimental studies have shown that *A. vasorum* isolates can readily be exchanged between dogs and red foxes by snails and frogs [1, 7] and field surveys have demonstrated that in foci with endemic infections in dogs also have high prevalences (up to 50% and more) in wild red foxes [13–16]. In addition, genetic analyses identified shared haplotypes between different definitive hosts such as dogs, red foxes and coyotes [17], suggesting the important role of wildlife, particularly red foxes, in the epidemiology of the parasite.

Several studies in dogs have contributed with information on clinical, diagnostic, pathological and epidemiological aspects as well as response to treatment, e.g. [18–21]. In contrast, notwithstanding their important role as reservoir, little is known from red foxes. There are no reported clinical data from red foxes and even relatively large worm burdens have not been associated with emaciation in necropsied foxes [3, 14, 16]. *Post-mortem* examination of naturally infected dogs and red foxes reveal similar lung lesions with the most prominent finding being congested, firm lung lobes with yellow/greyish mottled discoloration associated with massive inflammatory verminous pneumonia [22–24].

Studies dealing with the basic population biology of the parasite are few, e.g. [25]. The relationship between infection dose and establishment of worms as well as issues like age-related resistance and acquired immunity have not yet been addressed. These factors are fundamental for knowledge on the dynamics of infections in red foxes and dogs and imperative to evidence-based control; they are most appropriately investigated by means of experimental infections. The objective of the present study on red foxes was to investigate the impact of host age and inoculation dose on larval excretion, establishment of adult worms and selected clinico-pathological parameters carried out as a 2 by 2 factorial study. We hypothesized that juvenile red foxes, as compared to adults, given a high inoculation dose would be more susceptible to the infection and associated pathophysiology.

## Methods

### Experimental design and treatments

Thirty-four female red foxes (*Vulpes vulpes*) in two age groups, juveniles (5 months of age, *n* = 17) and adults (> 1.5 years, *n* = 17) were obtained from a fur farm. Within each age group, seven randomly selected red foxes received a single dose of 50 *A. vasorum* L3 (Groups Juv50 and Ad50) and another seven received 200 L3 (Groups Juv200 and Ad200). Prior to inoculation by a stomach tube, all animals were anaesthetised by intra-muscular injection of Zoletil®50 (a mixture of zolazepam; 5 mg/kg body weight, BW and tiletamin; 5 mg/kg BW; Chemvet) in combination with medetomidin (80 μg/kg BW, Domitor®Vet, Orion Pharma). Three additional juveniles (Group JuvC) and three adults (Group AdC) were simultaneously inoculated with tap water as uninfected controls. All 34 animals were euthanised 9 weeks post-inoculation (wpi). To follow L1 larval excretion over a longer period of time, an additional single (donor) fox (6–7 months of age) inoculated with 200 L3 was kept for 340 days post-inoculation (dpi). The study was performed under the Danish experimental animal licence no. 2005/561-1060.

### Experimental animals and sampling

The red foxes were housed individually in elevated cages in the university experimental animal facilities. Three weeks prior to inoculation, all foxes were treated with fenbendazole (50 mg/kg BW/day orally for 3 days, Panacur®). Faecal examination by a modified Baermann method [26, 27] confirmed that they were free from metastrongyloid infections prior to study start. Foxes were fed daily with 300–350 g of a commercial wet feed based on chicken, fish and plant material with energy content of 1,900 kcal/kg and they had access to water *ad libitum*. Blood samples were collected in serum, citrate and EDTA tubes weekly throughout the experiment from the external jugular vein and animals were weighed weekly 3–9 wpi (due to technical problems weighing week 0–2 was not performed). Clinical signs and well-being were monitored daily during feeding, and animals were examined more thoroughly at blood sampling. Faecal larval excretion in the fox with long-term infection was analysed 3 times weekly up to 200 dpi and subsequently once weekly up to 340 dpi.

### Parasite isolates

*Angiostrongylus vasorum* L1 obtained from faeces of a naturally infected dog were used to infect laboratory aquatic snails (*Biomphalaria glabrata*). Infection of snails were established by placing 5–6 snails in small containers with 100–200 L1 per snail in 20 ml tap water (enough to cover the snails) for 24 h under a constant light source. The snails were subsequently transferred to larger water tanks and kept there for 6 weeks. Infective L3 were obtained by tissue digestion: the snails were crushed and digested in 1% pepsin (1:10,000 IU) dissolved in 1% HCl (37%) in tap water at 37 °C for 10–20 min on a magnetic stirrer (modified from [28]). The fluid was then passed through a 180 μm sieve and allowed to settle for 30 min before the supernatant was discarded and the sediment washed 2–3 times in tap water.

The recovered L3 were used to inoculate four farm-bred red foxes becoming chronically infected and thus maintain a supply of L1 for further snail infections and production of L3. Finally, prior to inoculation of the experimental red foxes, the number of L3 in a subsample was counted and individual inoculation doses were prepared.

### Larval excretion

Fresh faecal samples were collected either from the cages or from the floor just beneath the individual cages from 28 dpi and subsequently 3 times weekly until necropsy. Samples were processed within 24 h after deposit using a modified Baermann method (*c*.10 g of faeces) and the sediments were transferred to tubes which were examined for L1 within a week. The number of L1 per gram of faeces (lpg) was determined.

### Haematology, blood biochemistry and coagulation profiles

Sera and plasma were isolated by centrifugation and kept at -20 °C until use. Standard haematology [total white blood cell count (WBC); leukocyte differential count; total red blood cell count (RBC); haematocrit; haemoglobin; and platelet count (PLT)] were performed using an ADVIA120 haematology analyzer (Siemens) using canine settings and a full biochemical profile including glucose, alanine-aminotransferase (ALT), fructosamine, total protein, total bilirubin, alkaline phosphatase (ALP), cholesterol, urea, creatinine, inorganic P, albumin, amylase, lipase, Na, K and Cl, were performed using an automated spectrophotometer (ADVIA 1650, Bayer Health Care Diagnostics, Berlin, Germany). All analyses were subjected to daily internal and quarterly external quality control, and only results from accepted runs were released. Coagulation analysis was performed from citrated plasma samples at 0, 2, 3, 6 and 9 wpi. Fibrinogen, prothrombin time (PT) and activated partial thromboplastin time (aPTT) were measured using an automated haemostasis analyzer (ACL9000, Instrumentation Laboratory). The concentration of D-dimer was measured using an immunometric flow-through principle (NycoCard READER II, Medinor). Normal dog plasma (pool of 5–10 dogs) was run as internal test control.

#### *Post-mortem* examination

A reverse perfusion of the cardio-pulmonary vascular system was developed for the study to improve the recovery of intact live worms at necropsy. Anaesthetized red foxes (see above) were given heparin intravenously (350 IU/kg BW) in order to prevent blood clotting during the following procedures. Three minutes later a lethal pentobarbital dose (100 mg/kg BW intravenously) was given, thorax was opened and thoracic organs were perfused *in situ* after aorta, vena azygos and both venae cavae were clamped off. About 3 l of isotonic perfusion fluid (15 g sodium citrate + 8.6 g NaCl dissolved in 1 l tap water) were pumped through a 16G needle into the left auricle, through pulmonary veins, lung capillaries and pulmonary arteries to the pulmonary trunk from which it was led *via* a plastic cylinder (5 mm in diameter) onto a 200 μm sieve for collection of worms. Recovered worms were directly transferred to RPMI 1640 medium until counting. The perfusion procedure was followed by removal of lungs and heart, dissection of both ventricles and pulmonary arteries and slicing of the lung tissue with fine scissors to obtain worms captured in nodules. Finally, the finely sliced lung tissue was placed floating in normal saline (baermannization) wherefrom additional sedimented worms were collected the following day. The worms were counted and determined to sex under a dissection microscope (×40).

### Statistical analyses

Parasitological data were analysed by two-way analysis of variance (ANOVA) with Bonferroni *post-hoc* tests with the two main factors age (juvenile/adult) and dose (high/low) and their interaction, and BW at week 3 as a covariate using SAS 9.0 (SAS Institute Inc., USA). Parameters were tested for normality before analysis. All blood parameters were analysed for each age group (juvenile or adults) separately, and reference ranges (means ± 2 SD) were established by using values of all animals from week 0 (*n* = 17 for each group). Animals of the infected groups and of the uninfected control group were compared by repeated measures ANOVA, with BW as a covariate. If overall significant, univariate tests for weeks were performed using Bonferroni multiple comparisons adjustment. In a few cases where a single blood value was missing due to sampling or assay error, it was constructed as the mean of the previous and next sample values. For platelets the repeated measures analysis excluded the first 3 weeks. Values with *P* < 0.05 were considered statistically significant.

## Results

### Clinical recordings and performance

Vomiting or any other adverse effects were not observed following inoculation of L3. No animals demonstrated any clinical signs of respiratory or cardio-vascular disease or any other kind of symptoms related to experimental infections during the study period. A single fox was treated with antibiotics due to otitis externa at 7 wpi. Mean live weights at 3 wpi were not significantly different (Table 1) and weight gains (approx. 1.5 kg) did not differ for infected groups compared to uninfected age controls (data not shown).

**Table 1:** Live weights and *ante mortem* parasitological data of red foxes (juveniles and adults) infected with 50 or 200 infective third-stage larvae of *Angiostrongylus vasorum*, including uninfected controls

Group	*n*	Mean ± SD live weight 21 dpi (kg)	Mean accumulated larval counts 41–63 dpi (range) (L1/g)*	Prepatent period (range of group) (days)
Juv50	7	5.2 ± 0.9^a^	62 (13–183)^a,b^	41–48
Juv200	7	5.3 ± 0.3^a^	150 (35–432)^b^	41–48
Ad50	7	6.2 ± 0.4^a^	53 (2–156)^a,b^	48–55
Ad200	7	6.1 ± 0.7^a^	4 (0–16)^a^	48–57^#^
JuvC	3	5.0 ± 0.2^a^	–	–
AdC	3	5.7 ± 0.2^a^	–	–

Numbers in columns with different superscripts are significantly different

*Abbreviations: dpi* days post-inoculation, *SD* standard deviation

*Statistical analysis by ANOVA on larval counts (*r* ^2^ = 0.38): Age: *F*_(1,23)_= 8.39, *P* = 0.0081; Dose: *F*_(1,23)_= 0.63, *P* < 0.44; Age*dose: *F*_(1,23)_= 5.94, *P* = 0.023; Groups were significantly different at *P* < 0.05 by Bonferroni post-hoc tests

^#^Two red foxes harbouring 2 and 23 worms did not reach patency

### Faecal excretion of first-stage larvae and worm counts

**Fig. 1:**
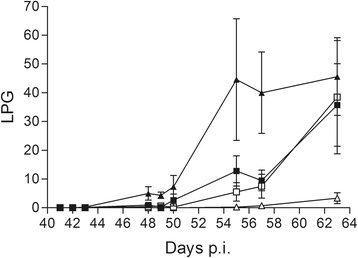
Mean faecal larval excretion of four groups of red foxes experimentally infected with *Angiostrongylus vasorum*. Mean (± standard error) first-stage larvae per g of faeces (lpg) from 41 days post-inoculation (p.i.) from foxes (*n* = 7 in each group) in groups Juv50 (***■***), Juv200 (***▲***), Ad50 (***□***), Ad200 (Δ). *Note*: Juv, juvenile foxes; Ad, adult foxes; 50/200, animals inoculated with 50 or 200 infective third-stage larvae, respectively

**Fig. 2:**
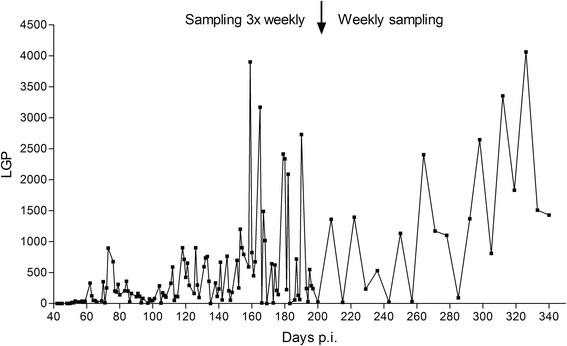
Faecal larval excretion pattern from one red fox single-infected with *Angiostrongylus vasorum.* Number of first-stage larvae (L1) per g of faeces (lpg) 42–340 days post-inoculation (p.i.) from a juvenile red fox orally infected with 200 third-stage larvae. Faecal samples were collected three times weekly up to 200 dpi and once a week thereafter

In the juvenile red foxes, first excretion of larvae was observed 41 dpi (week 6) and 48 dpi all had patent infections (Table 1). In contrast, the first adult foxes in both groups began excretion at 48 dpi (week 7) with all animals in group Ad50 reaching patency 55 dpi whereas two foxes in group Ad200 never reached patency. Excretion of L1 varied considerably from day to day in individual animals in all groups, and the highest individual output of 164 lpg was observed in group Juv200 at 55 dpi. At necropsy two red foxes in Ad50 had excretion of 99 and 127 lpg while the rest were below 25 lpg. For the mean larval counts a significant interaction of age and infection dose was found, indicating higher larval counts with higher dose in juveniles while the opposite was found in adults (Table 1; Fig. 1). The group Juv200 had higher larval output over time (41–63 dpi) than the Ad200 group (*t*_(23)_ = 3.64, *P* = 0.0082) (Table 1). Monitoring of the extra (donor) animal followed for almost a year demonstrated clearly the large variation in excretion between samplings, a high degree of persistence and lastly, markedly higher levels (up to 4000 lpg or 100× more) after the first 100 days (Fig. 2). Specimens of the intestinal trematode *Cryptocotyle lingua* was detected occasionally in faeces from foxes in different groups.

**Fig. 3:**
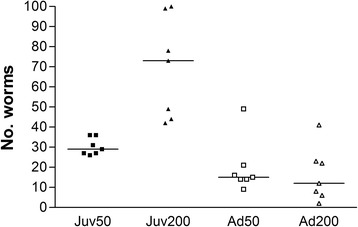
Individual *Angiostrongylus vasorum* worm counts ten weeks post-inoculation of red foxes (*n* = 7) in groups Juv50 (*■*), Juv200 (▲), Ad50 (*□*), Ad200 (∆). Medians are shown as horizontal bars. Group Juv200 differed significantly from the other three groups by Bonferroni *post-hoc* test (*P* < 0.05). *Note*: Juv, juvenile foxes; Ad, adult foxes; 50/200, animals inoculated with 50 or 200 infective third-stage larvae, respectively

Adult *A. vasorum* were recovered from all infected animals at necropsy (9 wpi) (Fig. 3) including the two adult foxes without L1 excretion. For mean worm counts, a highly significant interaction of age and infection dose was found, indicating higher worm counts with higher dose in juveniles but not in adults (Table 2). Significantly more worms were recovered from group Juv200 than the three other groups (*t*_(24)_ = -4.62, *P* = 0.0007; *t*_(24)_ = 5.88, *P* <0.0001; *t*_(24)_ = 6.28, *P* <0.0001 for groups Juv50, Ad50 and Ad200, respectively) (Fig. 3). The mean recovery of worms ranged from 8–61% in the different groups (Table 2). The overall proportions of worms recovered by the three different steps of the procedure (perfusion, dissection and Baermann method performed with sliced lung tissues) were 49%, 40% and 11% of the total worm counts, respectively. In contrast to dissection and the Baermann procedure, however, close to 100% of the worms recovered by perfusion were alive and intact. Due to damages during processing, 11% of the worms could not be determined to gender. No *A. vasorum* worms or larvae were recovered from the control foxes.

**Table 2:** *Post-mortem* worm counts and worm recovery (%) in red foxes (juveniles and adults) infected with 50 or 200 infective third-stage larvae of *Angiostrongylus vasorum*, including uninfected controls. Worm isolation comprised three subsequent procedures (reverse perfusion, dissection and baermanization of the lungs). Statistical analysis (ANOVA) included infected groups only

Group	*n*	Contribution to worm count (% of total)			Mean worm count (95% CI)	% female worms	Worm recovery % (range)
		Perfusion	Dissection	Baermann			
Juv50	7	57	32	11	30.3 (26.4–34.2)	50	61 (52–72)
Juv200	7	40	49	11	69.3 (46.3–92.3)	47	35 (21–50)
Ad50	7	49	36	15	19.7 (7.3–32.1)	50	39 (18–98)
Ad200	7	70	20	10	16.3 (3.8–28.7)	59	8 (1–21)
JuvC	3	–	–	–	0	–	–
AdC	3	–	–	–	0	–	–

*Abbreviation*: *CI* confidence interval

Statistical analysis on worm counts (*r* ^2^ = 0.68): Age: *F*_(1,24)_ = 28.39, *P* < 0.0001; Dose: *F*_(1,24)_ = 8.89, *P* =0.0065; Age*dose: *F*_(1,24)_ = 12.64, *P* = 0.0016

### Haematology and blood biochemistry

**Fig. 4:**
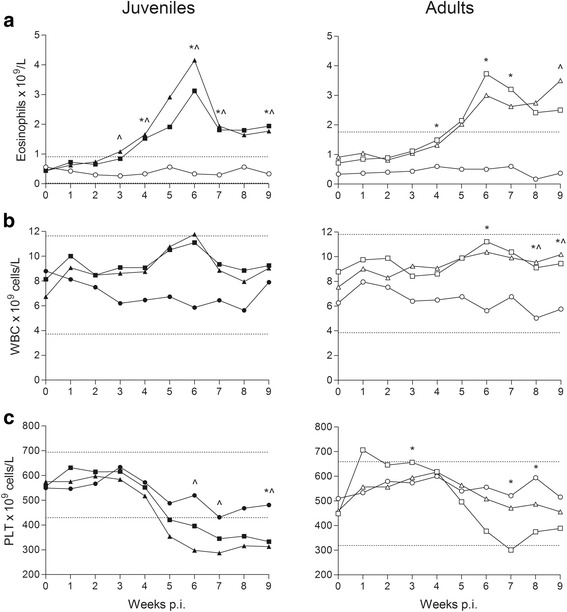
Mean haematological parameters from red foxes experimentally infected with *Angiostrongylus vasorum* and uninfected controls 0–9 weeks post-inoculation (p.i.). Mean counts of peripheral blood eosinophils (**a**), total white blood cells (WBC) (**b**) and platelets (**c**) in foxes during experimental *Angiostrongylus vasorum* infection. Groups: Juv50 (■), Juv200 (▲), JuvC (●) (controls), Ad50 (***□***), Ad200 (Δ), and AdC (○) (controls). Calculated reference ranges are indicated by horizontal dashed lines; only upper limit visible for eosinophils. Significant differences between infected and control groups are marked for dose 50 (*) and (^) for dose 200 at *P* < 0.05. *Note*: Juv, juvenile foxes; Ad, adult foxes; 50/200, animals inoculated with 50 or 200 infective third-stage larvae, respectively

Mean blood eosinophil counts were significantly elevated in infected compared to control groups (Juveniles: *F*_(1,14)_ = 16.56, *P* = 0.0011; Adults: *F*_(1,14)_ = 10.59, *P* = 0.0058); values out of range were seen 3 wpi in juveniles and 4 wpi in adults (Fig. 4a). Mean counts peaked around patency at 6 wpi for groups Juv50, Juv200 and Ad50 and 9 wpi for Ad200 but despite a decline, counts remained elevated for the rest of the study period. The mean WBCs were generally higher in infected compared to control groups although only significantly so for groups Ad50 and Ad200 *vs* AdC (*F*_(2,13)_ = 7.62, *P* = 0.0065) (Fig. 4b). The elevated WBCs probably reflected higher levels of eosinophils as analysis of other blood cell types did not reveal any increase.

**Fig. 5:**
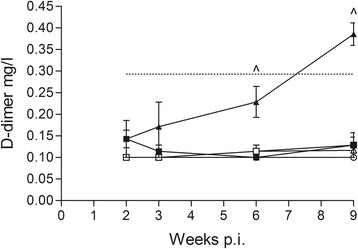
Mean (± standard error) values of D-dimer (mg/l) at 2, 3, 6 and 9 weeks post-inoculation (p.i.) in red foxes experimentally infected with *Angiostrongylus vasorum* and uninfected controls. Groups: Juv50 (■), Juv200 (▲), JuvC (●) (controls), Ad50 (□), Ad200 (∆), and AdC (○) (controls). Upper reference range for juvenile foxes are indicated by horizontal dashed line. All adults had 0.1 mg/l at 2 weeks p.i. and reference range could not be constructed. *Note*: Juv, juvenile foxes; Ad, adult foxes; 50/200, animals inoculated with 50 or 200 infective third-stage larvae, respectively

The numbers of blood platelets decreased over time in groups Juv50, Juv200 and Ad50 compared to controls, although not significantly when analysing the whole experimental period (Fig. 4c). Numbers in infected juveniles were below reference range from 5 wpi. By excluding the first three weeks after inoculation (due to low level of variation) from the repeated measures analysis, platelet numbers differed significantly between groups Juv200 and JuvC from 6 wpi and between Juv50 and JuvC at the end of the experimental period, with platelets being lower in Juv50. For the adult red foxes, values were significantly lower in group Ad50 than in group AdC 7 and 9 wpi. The concentration of D-dimer, indicative of active coagulation and fibrinolysis, was significantly increased in group Juv200 compared to both JuvC and Juv50 6 and 9 wpi (Fig. 5).

Serum albumin and total protein levels showed variation, and it was difficult to describe consistent differences between groups. However, mean serum albumin levels were significantly lower (8–15%) in infected adults compared with controls on several occasions after 2 wpi (*F*_(2,13)_ = 11.15, *P* = 0.0015) (data not shown). No significant differences between infected and uninfected groups were found for any of the remaining haematological and biochemical parameters, including fibrinogen, PT and aPTT.

## Discussion

### Age-related resistance

This study is the first to report on the influence of host age and infection dose on population dynamics of *A. vasorum* in red foxes, aspects that are fundamental for a better understanding of the role of red foxes as wildlife reservoir for this parasite, and may serve as model for infection dynamics in dogs. Juvenile red foxes (5 months old) were highly susceptible to a primary, single inoculation with *A. vasorum* whereas adult red foxes had significantly lower worm counts at the same dose level. This suggests an age-related resistance to infection. Age resistance, the fact that older animals become more resistant to a primary infection, is commonly recognised in ascarid infections, e.g. *Toxocara* spp. and *Ascaris suum* [29] but to our knowledge, this has not been reported for metastrongyloids previously. The significant interaction between age and dose level was the result of a clear dose-response in juveniles (high dose leading to higher worm counts) and a lack of such dose-response in adults (mean worm counts of 20 and 16 after inoculation of 50 and 200 L3/animal, respectively). Assuming increased susceptibility of young foxes and age-related resistance, such an interaction was anticipated. The establishment rates of adult worms were lower with increasing dose in both age groups. Similar pattern has been observed in foxes and dogs experimentally inoculated with *Toxocara canis* where extra-high inoculation doses (above 10,000 eggs) did not result in patent infection [30, 31]. Whether such an upper limit exists for the number of *A. vasorum* establishing in adult hosts or, whether it is linked to the experimental design with a single inoculation, needs further investigation. The variation in worm counts was remarkably large in adults inoculated with the low dose (18–98% recovery rates) which indicates that some adult red foxes remain highly susceptible despite age. This may eventually find support from epidemiological studies of wild red foxes. Intensity of *A. vasorum* infection in naturally infected foxes is variable and ranges from a mean of 6 worms in red foxes in Spain [32], 7.4–17.4 in Denmark [15, 16], 9.6 in Italy [33] and up to 72 in Newfoundland, Canada [14], with a single red fox harbouring 375 worms. Likewise, in dogs single observations revealed as many as 572 worms in a dog [34]. In highly endemic areas, higher prevalence was found with increasing age of the red foxes [15], although cubs had a trend (but not significant) towards higher worm burdens than adult or juvenile animals. In areas with low prevalence, a correlation between age and prevalence or worm burden could not be confirmed [3]. Also in a highly endemic area in Newfoundland, worm burden was unchanged with increasing age [14]. In this case, it was hypothesised that due to the fact that once established, adult worms may survive in dogs for years [35, 36], worms could have been established earlier in life also in red foxes. It was suggested that an immune response might limit the number of *A. vasorum* nematodes to a “threshold” intensity [14]; however this still needs to be substantiated. Interestingly, cubs of red foxes aged about a month, harboured worms in a Danish survey [15]. The latter is indeed surprising considering the minimum prepatency in this study of 40 days and the fact that cubs are not weaned until approximately eight weeks of age [37]. Although uncertainties with the age determination may account for 2–3 weeks variation, the reason for this unusual finding is not (yet) explained. In Spain, Mañas et al. [32] did not find *A. vasorum* in 2–3 month-old juveniles at necropsy.

The pattern of excretion of larvae largely substantiated above findings regarding susceptibility: larval numbers increased with increasing dose in juveniles whereas the opposite was the case for adult red foxes. In the adults inoculated with the high dose, the low larval excretion (only 2–3% of the larval excretion of the juveniles inoculated with the high dose), the presence of non-patent animals, and the longer pre-patent periods most likely reflect suppression of infections. Such reduced reproductive potential of *A. vasorum* in adult red foxes may imply that juvenile animals contribute relatively more to environmental contamination with L1. The uniform proportion of egg producing females (approx. 50% female worms in all experimental groups) indicate that within the study period such host age-related difference is not related to a skewed sex ratio of worms.

Although based on a single animal observation, it was truly remarkable that larvae excretion in a juvenile fox followed 340 dpi, reached up to a hundred times more than the average of any experimental group. It supports observations that persistent infections may be associated with very long, perhaps larval excretion for several years, even without reinfections [6, 25]. Larval excretion in this fox was considerably higher than in experimentally infected dogs, although increase after 150 dpi has also been observed in dogs recieving only a single inoculation [25]. Within the nine weeks duration of the present study, no correlation was found between numbers of adult worms and larval counts on the last sample day (or mean of the last three samples days), except for the adult red foxes inoculated with 200 L3 group in which the low larval output was positively correlated to their low worm counts. Based on flotation of dead L1 from frozen faecal samples, no correlation between worm and larval counts could be found in naturally infected red foxes [14]. Highly fluctuating larval excretions in dogs have been reported by others in both experimental and natural infections [25, 38, 39] and are probably related to intermittent egg production in adults as suggested in the literature [40].

In summary, the worm and larval data of the present experiment appear comparable to the levels demonstrated in naturally infected red foxes, where exposure presumably is continuous during most part of the year. In nature, the accumulated number of L3 ingested by red foxes over time is obviously not known. Althogh naturally infected gastropods (snails and slugs) have been shown to harbour up to approximately 300 L3 [28], studies from Denmark and the UK found the majority of these to harbour less than 50 larvae [28, 41]. Although administered as a single exposure, the inoculation doses used in the present study is equivalent to ingestion of 1–20 infected gastropods by a red fox, which may be a realistic assumption. It may also suggest that high worm burdens found in both young red foxes and dogs results from a gradual build-up over time, allowed by a relatively limited protective immunity, as demonstrated in this study. Individual dogs may adopt behaviour of eating gastropods selectively [42] but accidental ingestion of smaller individuals through grass eating [43, 44] or stick transports [45], common phenomena in dogs, may offer a more likely explanation for infection in a larger proportion of dogs. Red foxes are also reported to occasionally prey on gastropods although such behaviour is related to season and age, e.g. with cubs rarely eating snails or slugs [37]. Lastly, we need to emphasize that other means of infection than intake of snails cannot be ruled out. Spontaneous shedding of L3 in mucus from snails was recently demonstrated for several metastrongyloid parasites, including *A. vasorum* [46, 47]. Such L3 may remain viable for some time in the environment depending on temperature and humidity. For *A. cantonensis* a longevity of up to 72 h has been estimated for L3 having merged from land snails [48].

### Clinical observations and morbidity markers

Well-known signs of clinical canine angiostrongylosis such as coughing, dyspnoea or bleeding abnormalities were not observed at any time during our study. There may be several explanations for this: an inherent better adaptability of red foxes to infection (low pathogenicity), too low level of exposure, no reinfection or continuous exposure which is often the case during natural infections, or signs may have been observed if red foxes had been kept longer or under natural conditions where exercise was needed. However also in dogs, natural infections often go unnoticed [10, 49, 50] and clinical signs are not necessarily present in experimental infections [25, 51]. Acute signs like vomiting related to the inoculation are not uncommon [25, 51, 52]. The weight gains observed in both age groups of red foxes were related to season and were comparable between infected and control groups, indicating that the induced infection levels did not have a noticeable impact on voluntary feed intake or feed utilisation. Similar conclusions were made for experimentally infected dogs [51], but also the opposite was observed [11]. In a few cases *C. lingua* were found in faecal samples from the red foxes. Although *C. lingua* is common in wild red foxes in Denmark [15], infection in the present experimental animals indicates that the parasites were most likely introduced by feed containing fish material without heat treatment [53]. Potential interactions between *C. lingua* and *A. vasorum* cannot be ruled out.

Different disease markers have been evaluated in the pursuit of early and safe diagnosis of *A. vasorum* infection but even though a range of haematological and biochemical indicators and coagulation profiles may support the diagnosis performed by faecal Baermann analysis or PCR [35, 54], none are specific or conclusive [9, 11, 19, 20]. Serological tests with high specificity and sensivity for *A. vasorum* antigen and antibody detection are in use for dogs [55–58], but have not yet been evaluated for red foxes.

The relatively short duration (9 weeks) of our study and the low inoculation doses may explain the limited changes in certain blood parameters. Furthermore, elaboration on the relative importance of differences between infected and control groups was inconclusive as only internal reference ranges for red foxes were available. Nevertheless, it was apparent that infections were accompanied by marked eosinophilia, peaking at the time of patency 6 wpi, and a simultaneous thrombocytopenia. The eosinophilia observed in both age groups and doses was also reflected in a transitory leukocytosis. Eosinophilia (and leukocytosis) has been reported in naturally infected dogs [9, 21] and in experimental infections around 3–7 wpi [11, 18, 51]. Pronounced neutrophilia has also been reported in infected dogs [9, 11, 18] but this was not confirmed in our study or in a recent study in dogs [21]. Platelet numbers in red foxes were generally higher than in dogs (300–700 × 10^9^/l in red foxes as compared to our laboratory reference range of 200–500 × 10^9^/l in dogs) but levels in juveniles went below the calculated reference range and showed a significant dose dependency. The decline in platelet numbers (about 40%) and the time of onset around 3–4 wpi correspond again well to observations in experimentally infected dogs [18]. Changes in blood proteins were variable and inconsistent. A decrease in serum fructosamine in a normoglycaemic dog may indicate disturbance in protein metabolism. In contrast to findings in dogs with natural *A. vasorurm* infection [20], decreased fructosamine was not observed in our study.

Limited studies encompass coagulapathies of red foxes and we applied a range parameters previously used for dogs [10]. Apart from the mentioned decrease in platelet numbers, the main finding was a continuous and significant increase in D-dimers in the high dose juvenile group, a measure of degradation of cross-linked fibrin, which together with thrombocytopenia may reflect disseminated intravascular coagulation (DIC) in young red foxes. Similarly, thrombocytopenia and increase in D-dimers (> 4-fold) were seen in two fatal cases of cerebral haemorrhage in naturally infected dogs [59]. Since only the highest dose group amongst juveniles showed any increase, a dose dependency is evident in D-dimers levels in the acute phase of *A. vasorum* infection in fully susceptible foxes. Whether DIC would have resulted in hemorrhagic diathesis later on, remains speculative. Measurements of other coagulation parameters, PT and aPTT, were performed but data were not reliable, most likely due to prolonged enzyme activity after blood sampling (in opposition to D-dimers, which are not enzyme activity related). In experimentally infected dogs, these parameters were within reference ranges for up to 12 wpi [11]. A tendency to more severe pathophysiological changes in juveniles is in accordance with observations that dogs presenting with clinical signs are most often young [10, 60].

### Critical view on methodology

Quantification of adult *A. vasorum* is not trivial because in dead hosts the majority of adult worms are no longer located in the heart but in the peripheral branches of the pulmonary arteries and thus, not easily accessible. Dissection and rinsing of the lung tissue and heart are the gold standard for *A. vasorum* isolation from wildlife or dogs found dead [3, 4, 14]. They give a rough estimate of worm burden but frequently result in damaged worms and potential difficulty for differentiation from other lungworms. The developed reverse perfusion technique including injection of heparin (to prevent blood clotting in small vessels) prior to euthanasia and perfusion immediately after death offers great advantages for facilitated counting and for isolating live, intact worms for e.g. worm cultures. The recovery by perfusion was remarkably high in the adult high dose group. This may possibly indicate a shift in location with age and/or a different location in “resistant” animals. Nevertheless, subsequent dissection and baermanization/sedimentation regularly revealed numerous additional worms in the lung vessels. Especially male worms were recovered by the sedimentation method - most likely because they are more slender and transparent and thus more difficult to recognize by dissection. It is therefore recommended to apply the reverse lung perfusion in combination with traditional dissection methods for research purposes.

## Conclusions

The present study has demonstrated that establishment of a primary *A. vasorum* infections in red foxes is related to host age and inoculation dose. Natural resistance is generally higher in older animals, and this age resistance is reflected in lower worm burdens and much lower larval excretion. However, some old animals showed only limited age resistance. In the fully susceptible juvenile red foxes, a higher dose resulted in higher worm burdens, higher larvae excretion and more pronounced pathophysiological changes, particularly in coagulation parameters. Furthermore, infections seem to be chronic. The gradual increase in larval excretion after several months in a single animal, indicated that any potential acquired immunity does not affect worm fecundity. The findings in red foxes resemble to a large degree findings in dogs, and we hypothesize that the dynamics of the parasite population in the present experimental study, may not only contribute to our knowledge on the transmission among the wildlife reservoirs and between these and dogs, but also provide better understanding of infections in dogs.

## Abbreviations

ALP: Alkaline phosphatase
ALT: Alanine-aminotransferase
ANOVA: Analysis of variance
aPTT: Activated partial thromboplastin time
BW: Body weight
Dpi: Days post-inoculation
EDTA: Ethylenediaminetetraacetic acid
L1: First-stage larvae
L3: Third-stage larvae
Lpg: L1 per gram of faeces
PLT: Platelet count
PT: Prothrombin time
RBC: Red blood cell count
WBC: White blood cell count
Wpi: Weeks post-inoculation
